# Improving prevention and care for HIV and sexually transmitted infections among men who have sex with men in Cambodia: the sustainable action against HIV and AIDS in communities (SAHACOM)

**DOI:** 10.1186/s12913-016-1857-9

**Published:** 2016-10-21

**Authors:** Siyan Yi, Sovannary Tuot, Pheak Chhoun, Khuondyla Pal, Chanrith Ngin, Sok Chamreun Choub, Carinne Brody

**Affiliations:** 1 KHANA Center Population Health Research, No. 33, Street 71, Tonle Bassac, Chamkar Mon, Phnom Penh, Cambodia; 2 Center for Global Health Research, Public Health Program, Touro University California, 1310, Club Drive, Vallejo, CA94592 USA

**Keywords:** Men who have sex with men (MSM), HIV, Sexually transmitted infections (STIs), Community-based interventions, Sustainable Action against HIV and AIDS in Communities (SAHACOM), Cambodia

## Abstract

**Background:**

The prevalence of HIV and sexually transmitted infections (STIs) among key populations in Cambodia continues to rise. To address this issue, KHANA, the largest national HIV organization in the country developed and implemented the Sustainable Action against HIV and AIDS in Communities (SAHACOM) project. This study aims to determine the impacts of the SAHACOM on sexual behaviors and the uptake of HIV/STI services among men who have sex with men (MSM) in Cambodia.

**Methods:**

We compared outcome indicators at midterm (*n* = 352) and endline (*n* = 394). Surveys were conducted in 2012 and 2014 in Battambang and Siem Reap provinces. A two-stage cluster sampling method was employed to select the study sample for structured interviews.

**Results:**

The midterm and endline samples were similar. The average number of sexual partners in the past three months decreased significantly from 6.2 to 4.0 (*p* = 0.03). The proportion of MSM who reported paying for sex with men in the past three months also decreased significantly from 19.0 % to 9.7 % (OR = 2.0, 95 % CI = 1.3-3.0). No significant change was found in condom and lubricant use in all types of relationships. Regarding STIs, 28.1 % of MSM at midterm reported having at least one STI symptom in the past three months compared to 6.1 % at endline (OR = 4.6, 95 % CI = 2.9–7.4); out of them, 14.1 % of MSM at midterm sought treatment compared to 20.7 % at endline (OR = 2.6, 95 % CI = 1.1–6.9). The proportion of MSM who reported using illicit drugs in the past three months also decreased significantly from 12.2 % to 5.1 % (OR = 2.4, 95 % CI = 1.4–4.2). However, the proportion of MSM who reported having been tested for HIV in the past six months decreased significantly from 94.1 % to 77.1 % (OR = 2.9, 95 % CI = 1.8–3.6).

**Conclusions:**

Findings from this study indicate that the SAHACOM was effective in improving sexual behaviors and related health outcomes among MSM under the project. However, it could not increase condom use and HIV testing rates among this key population. Tailored intervention programs are needed to improve condom use and HIV testing among MSM in Cambodia.

**Electronic supplementary material:**

The online version of this article (doi:10.1186/s12913-016-1857-9) contains supplementary material, which is available to authorized users.

## Background

While the prevalence of HIV and sexually transmitted infections (STIs) in the general population has declined across the globe, the prevalence among men who have sex with men (MSM) continues to rise [1, 2]. Research has demonstrated that MSM have a higher risk of being infected with HIV and STIs compared to men in the general population [1, 3–5]. MSM are considered a hard-to-reach population due to the multiple levels of complex individual, social, and structural factors that prevent them from seeking HIV and STI services [6, 7]. MSM’s sexual behaviors overlap with homosexual, bisexual, and commercial behaviors, which pose even greater challenges for prevention of HIV and STIs in this key population [8]. Community-based intervention models have been tested and proved to be effective in improving sexual and reproductive health outcomes among MSM in different settings [8–12]. Despite these successes, challenges remain [8, 10].

Cambodia has recently been lauded for its success in slowing down their national HIV epidemic. HIV prevalence in the general population had fallen from a peak of approximately 2.0 % a decade earlier to 0.6 % in 2013, and annual new HIV infections have dropped 20-fold over the same period [13, 14]. Annual AIDS-related deaths have also declined dramatically by two-thirds over the past 10 years [13–15]. However, the epidemic remains concentrated at a very high rate among key populations, including MSM [16, 17]. According to the BROS Khmer Study conducted in 2010, the prevalence of HIV among MSM was 2.2 %, and 42.9 % of them reported having experienced at least one STI symptom in the past 12 months [18]. Furthermore, only about half of MSM had been tested for HIV, and 14.2 % sought STI testing and treatment in the past year [18]. Intervention programs must tailor services to the needs of the key population in order to prevent a resurgence of HIV. High coverage of the access to care and treatment for people who are tested positive for HIV and STIs must be maintained and stigma and discrimination they face in communities and when accessing healthcare services must be reduced [14].

Starting in October 2009, KHANA, the largest national HIV organization in Cambodia, has implemented the Sustainable Actions against HIV and AIDS in Communities (SAHACOM) project. Funded by the United States Agency for International Development (USAID), the five-year project utilized a community-based approach to empower and create ownership in communities [16, 17, 19]. The SAHACOM consists of three main components [17]: (1) community- and home-based care for people living with HIV and orphans and vulnerable children through community support volunteers and self-help groups and livelihood and economic strengthening with support for access to health facilities; (2) focused prevention programs for key populations with a particular emphasis on under-served and marginalized groups, including the promotion of HIV and STI testing and linking HIV-positive people to treatment and care services; and (3) capacity building of national networks of implementing partners and self-help groups.

Specifically for MSM, KHANA works with two implementing partners providing a package of community-based activities. These activities include: (1) outreach and behavioral change communication that aim to increase awareness of risky sexual behaviors related to HIV and STI transmission; (2) distribution of free condoms and lubricant; (3) HIV and STI screening by peer counselors at the community level as an important entry point into care for MSM and their partners; (4) immediate enrolment in pre-antiretroviral therapy (pre-ART) and ART services for HIV-positive MSM; and (5) harm reduction intervention providing education on HIV and illicit drug overlapping risk behaviors [17, 19, 20].

The end-of-project evaluation was conducted to review the intervention activities and measure changes in terms of efficiency and effectiveness of the programs, and the extent to which the core objectives of the project had been achieved by comparing outcome indicators measured at midterm and endline. This paper summarizes the key findings of an evaluation of the impact of the SAHACOM on prevention and care for HIV and STIs among MSM under the project.

## Methods

### Study sites and population

The midterm survey was conducted in 2012 among 352 MSM, and the endline survey was conducted in April 2014 among 394 MSM in Battambang and Siem Reap provinces. Details of the main study have been published elsewhere [21–25].

### Sample size and sampling

We employed the z test for a two-sample comparison of proportions to detect a change of 10 % - 15 % of key indicators such as consistent condom use and HIV testing, with a power of 80 % and alpha of 0.05. Design effect of two was set to compensate the cluster effect. The minimum sample size required for the study was approximately 310. After adjustment for incomplete response and missing data of 10 %, the minimum required sample size was approximately 350.

To select the study samples, a two-stage cluster sampling method was used. The sample size was proportionally allocated to the size of MSM in each province. We included only Battambang and Siem Reap provinces because the number of MSM covered by the SAHACOM was too small in other provinces when the midterm survey was conducted. Furthermore, the total number of MSM population in these two provinces represented more than 70 % of the total number of MSM covered by the focused prevention programs of the SAHACOM.

We used communes in each province as the smallest unit for the sampling process. A commune would be included in the study if it was under the coverage of the SAHACOM programs for at least 12 months prior to the data collection. Entertainment venues or hotspots, where MSM congregated, were identified by our community-based implementing partners and/or generated by using geographical information system mapping previously developed by our research team. The venues or hotspots included nightclubs, discotheques, sport clubs, beauty salons, streets, parks, private houses, and specific communities. MSM were approached at the venues or hotspots, where face-to-face interviews were conducted. To be included in this study, a person must: (1) be aged 18 years or older; (2) self-report as an MSM; (3) have lived in a catchment area of the SAHACOM for at least 12 months as identified by outreach workers; (4) be able to provide consent to participate in the study; and (5) be able to present themselves on the day of the data collection. MSM who were mentally and/or physically too sick to participate were excluded from the study.

### Data collection training

All interviewers and field supervisors were trained for two days on data collection methods, and one day was allocated for tool pretesting and reflection. The training covered necessary skills including interview techniques, confidentiality, and privacy assurance. The study protocol and tools were also reviewed during the training sessions in order for the team members to be thoroughly familiar with them. We also included quality control skills such as rechecking and reviewing the questionnaires after administration as well as resolving issues that might arise during the fieldwork. Field supervisors were encouraged to perform regular sessions with interviewers during the survey period to review progress and communicate any issues occurring during the data collection.

### Questionnaire development

A structured questionnaire was initially developed in English and then translated into Khmer, the national language of Cambodia. Another translator back-translated it into English to ensure that the “content and spirit” of every original item were maintained. Clear instructions and explanations were addressed to avoid any confusion during the interviews. Standardized tools were adapted from previous studies in the same population [18, 26], the most recent Cambodia Demographic and Health Survey [27], and other studies in Cambodia [28–34]. For details of the questionnaire﻿, please see “Additional file 1.”

Prior to the data collection, a pilot study was conducted with 10 MSM in Phnom Penh to ensure that the wording and contents were culturally acceptable and clearly understandable for the study participants. Necessary modifications were made based upon feedback from the pilot study and comments from researchers and practitioners working on HIV among MSM in Cambodia before finalizing the questionnaire.

Socio-demographic characteristics included age, marital status, years of formal education completed, main occupation, average monthly income, living situations, and personal perception about gender identity. Yes/no questions were used to collect information on several sexual behaviors, including sexual activities and condom and lubricant use with different partners such as regular female partners, regular male partners, female sex workers, male sex workers, female clients, and male clients in the past three months. Respondents were also questioned about HIV-related education they received in the past six months, STI symptoms and healthcare seeking behaviors for the symptoms in the past three months, and illicit drug use in the past six months.

### Data entry and analyses

Epi Data version 3 (Odense, Denmark) was used for data entry into a computerized database, and SPSS version 22.0 (IBM Corporation, New York, USA) was used for all data analyses. Double data entry was performed to minimize entry errors. Descriptive statistical analyses were conducted to compute means and standard deviations (SD) for numerical variables and frequencies (%) for categorical variables. Chi-square test or Fisher’s exact test were used for categorical variables and paired Student’s *t*-test for continuous variables to compare socio-demongraphic characteristics of respondents and outcome indicators to detect changes from midterm to endline. Two-sided *p*-values <0.05 was used to indicate statistical significance.

## Results

### Socio-demographic characteristics

Comparisons of socio-demographic characteristics of MSM at midterm and endline are shown in Table 1. The mean age of respondents at midterm was 23.3 years (SD = 5.3 years) and at endline was 23.7 years (SD = 5.2 years). Levels of their formal education were also similar. The majority of them (>90 %) were never married, and more than 80 % lived with their parents or relatives. Working in a hairdresser’s or a beauty salon were the most common job reported in both surveys. Almost all respondents at midterm and endline reported living in their current city for over three years. Regarding their gender identity, a significantly higher proportion of MSM at midterm perceived themselves as women (39.2 % vs. 20.6 %, OR = 2.5, 95 % CI = 1.8–3.5), while a significantly higher proportion of MSM at endline perceived themselves as men (42.0 % vs. 57.8 %, OR = 1.9, 95 % CI = 1.4–2.5).

**Table 1:** Comparisons of socio-demographic characteristics of MSM at midterm (*n* = 352) and endline (*n* = 394)

Characteristics	Midterm	Endline	OR (95 % CI)
Mean age (in years)	23.3 ± 5.3	23.7 ± 5.2	0.84
Marital status			
Never married	323 (92.0)	355 (90.3)	1.2 (0.7–2.0)
Married	22 (6.0)	29 (7.4)	1.2 (0.7–2.1)
Divorced/separated	7 (2.0)	10 (2.3)	1.3 (0.5–3.4)
Mean years of formal education completed	9.6 ± 3.0	9.5 ± 3.2	0.92
Currently living with			
Parents/relatives	268 (76.2)	316 (80.4)	1.3 (0.9–1.8)
Spouse/sexual partner	34 (9.6)	32 (8.2)	1.2 (0.7–2.0)
Friends/colleagues	18 (5.0)	25 (6.4)	1.6 (0.9–2.7)
Alone	32 (9.2)	20 (5.0)	1.9 (1.0–3.3)
Major occupation			
Hairdressing and beauty salon	94 (26.7)	112 (28.6)	1.1 (0.8–1.5)
Students	88 (25.0)	102 (26.0)	1.0 (0.8–1.5)
Farmer or laborer	59 (16.7)	59 (15.1)	1.1 (0.8–1.7)
Self–employed	38 (10.9)	58 (14.1)	1.4 (0.9–2.2)
Office worker (private, NGO)	49 (13.9)	44 (11.4)	1.3 (0.8–2.0)
Other (including unemployed)	24 (6.8)	19 (4.8)	1.8 (1.0–3.9)
Duration living in the current city >3 years	328 (93.2)	360 (92.1)	1.3 (0.7–2.2)
Perceived gender identity			
Women	138 (39.2)	80 (20.6)	2.5 (1.8–3.5)
Men	148 (42.0)	228 (57.8)	1.9 (1.4–2.5)
Both sex	66 (18.8)	85 (21.6)	1.2 (0.8–1.7)

Values are number (%) with odds ratio (95 % CI) for categorical variables and mean ± standard deviation with p-value for continuous variables

*Abbreviations*: *CI* confidence interval, *MSM* men who have sex with men, *NGO* non-governmental organization; OR, odds ratio

### HIV testing and referral services

Table 2 shows the comparisons of HIV testing and referrals among MSM at midterm and endline. Lifetime HIV testing rate was not significantly different at midterm and endline (87.2 % vs. 83.0 %). However, the proportion of MSM reported having been tested for HIV in the past six months decreased significantly from 94.1 % at midterm to 77.1 % at endline (OR = 2.9, 95 % CI = 1.8–3.6). Facilities where MSM received their most recent HIV test also changed significantly. MSM at midterm were significantly more likely to have their most recent HIV test at a voluntary confidential counseling and testing center (74.9 % vs. 43.5 %, OR = 1.4, 95 % CI = 1.1–1.8 %), while MSM at endline were significantly more likely to receive it through community-/peer-initiated testing and counseling (18.6 % vs. 41.2 %, OR = 3.1, 95 % CI = 2.2–4.5). MSM at endline were also significantly more likely to report having their most recent HIV test at a private facility (2.9 % vs. 10.1, OR = 3.7, 95 % CI = 1.8–7.9). Regarding referrals for HIV testing, MSM at midterm were significantly more likely to be referred by outreach workers (75.9 % vs. 65.1 %, 95 % CI = 1.1–2.1) and less likely to be self-referred (11.7 % vs. 19.6 %, OR = 1.9, 95 % = 1.2–3.0). Majority of the respondents at both midterm and endline received the results and counseling for their most recent HIV testing and HIV-related education in the past 12 months.

**Table 2:** Comparisons of HIV testing and referral services among MSM at midterm (*n* = 352) and endline (*n* = 394)

HIV testing and referrals	Midterm	Endline	
	*n* (%)	*n* (%)	OR (95 % CI)
Ever been tested for HIV	307 (87.2)	326 (83.0)	1.4 (0.9–2.1)
Tested for HIV in the past 6 months	289 (94.1)	252 (77.1)	2.9 (1.8–3.6)
Place of the most recent HIV testing			
VCCT center	230 (74.9)	142 (43.5)	1.4 (1.1–1.8)
C/PITC	57 (18.6)	135 (41.2)	3.1 (2.2–4.5)
Private clinic/hospital	9 (2.9)	33 (10.1)	3.7 (1.8–7.9)
Other	11 (3.6)	17 (5.2)	1.5 (0.7–3.2)
Person who advised to get the most recent HIV testing			
Peer educator, NGO staff	233 (75.9)	213 (65.1)	1.5 (1.1–2.1)
Myself	36 (11.7)	64 (19.6)	1.9 (1.2–3.0)
Friends	36 (11.7)	34 (10.4)	1.1 (0.7–1.8)
Other	2 (0.7)	2 (0.6)	1.0 (0.1–7.3)
Received the last HIV test result	301 (98.1)	320 (98.2)	1.1 (0.3–3.3)
Received the counseling for the last test	286 (94.7)	312 (95.7)	1.6 (0.8–3.3)
Received HIV education in the past 12 months	292 (83.0)	333 (84.7)	1.1 (0.8–1.3)

*Abbreviations*: *CI* confidence interval, *C/PITC* community-/peer-initiated testing and counseling, *MSM* men who have sex with men, *NGO* non-governmental organization, *OR* odds ratio, *VCCT* voluntary confidential counseling and testing

### Sexual behaviors and condom use

As shown in Table 3, mean number of sexual partners in the past three months decreased significantly from 6.2 (SD = 12.4) at midterm to 4.0 (SD = 5.5) at endline (*p* = 0.03). The proportion of MSM who reported paying for sex with men in the past three months also decreased significantly from 19.0 % at midterm to 9.7 % at endline (OR = 2.0, 9 % CI = 1.3–3.0). However, a significantly higher proportion of MSM at end line reported selling sex to women in the past three months (4.8 % vs. 8.9 %, OR = 1.8, 95 % CI = 1.1–3.3), and having sexual intercourse with girlfriends in the past three months (15.6 % vs. 29.9 %, OR = 1.8, 95 % CI = 1.3–2.5) compared to MSM at midterm. No significant difference was detected in condom and lubricant use rates with all types of partners at midterm and endline.

**Table 3:** Comparisons of sexual behaviours and condom use among MSM at midterm (*n* = 352) and endline (*n* = 394)

Sexual behaviors	Midterm	Endline	OR (95 % CI)
Number of sex partners in the past 3 months	6.2 ± 12.4	4.0 ± 5.5	0.03
Paying for sex with women in the past 3 months	31 (8.8)	57 (14.5)	1.3 (1.0–2.6)
Always used condom when paying for sex with women	28 (90.3)	44 (77.2)	1.2 (0.6–2.2)
Sold sex to women in the past 3 months	17 (4.8)	35 (8.9)	1.8 (1.1–3.3)
Always used condom when selling sex to women	16 (94.2)	27 (79.4)	1.2 (0.5–2.8)
Had paying sex with men in the past 3 month	67 (19.0)	38 (9.7)	2.0 (1.3–3.0)
Always used condom when paying for sex with men	51 (73.9)	27 (73.0)	1.1 (0.6–2.0)
Sold anal sex to men in the past 3 months	69 (19.6)	67 (17.0)	1.2 (0.8–1.7)
Always used condom when selling anal sex to men	47 (68.1)	49 (73.1)	1.1 (0.6–1.8)
Always used lubricant when selling anal sex to men	34 (49.3)	41 (64.1)	1.2 (0.7–2.2)
Had sex with boyfriends in the past 3 months	223 (83.5)	206 (86.9)	1.2 (0.9–1.5)
Always used condom with boyfriends in past 3 months	138 (62.4)	134 (64.7)	1.1 (0.8–1.4)
Had sex with girlfriends in the past 3 months	59 (15.6)	118 (29.9)	1.8 (1.3–2.5)
Always used condom when having sex with girlfriend	37 (62.7)	65 (55.1)	1.1 (0.7–1.9)

Values are number (%) with odds ratio (95 % CI) for categorical variables and mean ± standard deviation with p-value for continuous variables

*Abbreviations*: *CI* confidence interval, *MSM* men who have sex with men, *OR* odds ratio

### STI symptoms, care-seeking behaviors, and illicit drug use

Comparisons of STI symptoms and illicit drug use among MSM at midterm and endline are shown in Table 4. Regarding STIs, 28.1 % of MSM at midterm reported having at least one STI symptom in the past three months compared to only 6.1 % among MSM at endline (OR = 4.6, 95 % CI = 2.9–7.4); out of them, only 14.1 % of MSM at midterm sought treatment for their most recent symptoms compared to 20.7 % among MSM at endline (OR = 2.6, 95 % CI = 1.1–6.9). The proportion of MSM who reported using any type of illicit drugs also decreased significantly from 12.2 % at midterm to 5.1 % at endline (OR = 2.4, 95 % CI = 1.4–4.2).

**Table 4:** Comparisons of STI symptoms and illicit drug use among MSM at midterm (*n* = 352) and endline (*n* = 394)

STI symptoms and illicit drug use	Midterm	Endline	
	*n* (%)	*n* (%)	OR (95 % CI)
Had at least one STI symptom in the past 3 months	99 (28.1)	24 (6.1)	4.6 (2.9–7.4)
Sought treatment for the most recent symptom	14 (14.1)	6 (20.7)	2.6 (1.1–6.9)
Facility where the most recent STI was treated			
Public health center/hospital	4 (28.6)	4 (66.7)	2.3 (0.4–12.6)
NGO clinic/hospital	9 (64.3)	2 (33.3)	1.9 (0.3–11.7)
Private clinic/hospital	1 (7.1)	0 (0.0)	1.3 (0.1–37.6)
Illicit drug use in the past 6 months	43 (12.2)	20 (5.1)	2.4 (1.4–4.2)

*Abbreviations*: *CI* confidence interval, *MSM* men who have sex with men, *NGO* non-governmental organization, *OR* odds ratio, *STI* sexually transmitted infection

## Discussion

This study shows significant changes in several key outcome indicators from midterm to endline. Significant impacts of the SAHACOM could be seen in the reduction of the number of sexual partners and paying for sex. The proportion of MSM who reported having STI symptoms decreased, while the proportion of MSM who reported seeking care for their most recent episode of STIs increased significantly. Illicit drug use also decreased significantly. However, negative changes should also be noted. These included the reduction in reported recent HIV testing rate and the increase in involvement in sex work and sexual intercourse with regular female partners. It is worth-noting that, although no significant change was found in condom and lubricant use in all relationships from midterm to endline, condom use with regular male partners increased dramatically from only 27 % at baseline in 2010 [20] to 62.4 % at midterm and 64.7 % at endline. However, while this information is useful for program implementers, this comparison must be made with caution given that the rate of condom use at baseline was from program data collected through desk reviews and consultative meetings with key stakeholders [20], and no details on the data collection methods are available.

The large change in condom use from baseline to midterm which was sustained to endline as well as the reduction in STI symptoms can be related to several components of the SAHACOM project including the community-based prevention and support approach, where community volunteers and outreach workers were able to reach mobile and hard-to-reach MSM. In addition, drop-in centers may have played an important role in conjunction with outreach workers by providing peer-support group discussions that increased knowledge about how to reduce risky behaviors. A pilot program in Cape Town, South Africa also demonstrated that community-based social activities and group meetings were viable strategies for disseminating HIV-prevention information, condoms, and lubricant to MSM [9]. Other studies also proved the effectiveness of using trained peers to provide rapid HIV and STI testing to MSM and link them to care in China [12] and Denmark [11].

This evaluation suggests that participation in the programs was associated with the reduction in the proportion of MSM who reported having been tested for HIV in the past six months from midterm to endline. The decline could possibly be explained by the budget shortages in year four of the SAHACOM, which resulted in deficiency of finger-prick testing materials and reduction in the number of community support volunteers and outreach workers. As afore-mentioned, the community workers were responsible for HIV and STI testing for MSM, educating them on HIV and sexual and reproductive health, and referring them to healthcare services [19]. High turnover rates due to low incentive had resulted in more time and efforts for recruitment and training of new outreach workers and slowed down the outreach activities, particularly community-/peer-initiated HIV testing and counseling. According to the SAHACOM program data, the number of MSM reached by the focused prevention program decreased from 5,020 in 2012 to 4,447 in 2013, and the number of HIV tests decreased from 3,692 to 2,660 over the same period. The shortfall in staff and supplies could also account for the lack of change in the reported condom and lubricant use from midterm to endline as the number of distributed condoms and lubricant decreased from 384,309 in 2012 to 270,393 in 2013.

The findings of the unappreciable improvement of the rates of condom use, coupled with the reduction in HIV testing, may also indicate that additional studies are needed to explore community-based approaches to improve and sustain regular HIV and STI testing and condom use among MSM in Cambodia. HIV testing and condom use are complex behaviors affected by a multitude of factors, including stigma and discrimination [35, 36]. A peer-led, community-based rapid HIV testing intervention [12] was able to circumvent barriers to HIV testing such as stigma and discrimination at health facilities, lack of awareness about where to get tested, and inconveniences, reported in previous studies [37–40]. Further studies should be conducted to understand the extent to which stigma and discrimination may affect HIV testing as well as condom and lubricant use among MSM in Cambodia, and broad community-based interventions are needed to reduce stigma and discrimination.

An important component of the SAHACOM that might be effective was HIV education, which was found to be linked to the increase in consistent condom use and HIV testing through partnership with MSM networks [14]. Working with MSM through MSM community networks was important to increase access to HIV education, HIV/STI testing, and condoms/lubricant for this population. Emphasis on consistent condom use with any types of sexual partners should be continued in future programs. The main sources of HIV and sexual and reproductive health education received by the respondents in this study were peer educators or outreach workers, demonstrating once again that the SAHACOM using the community-based approach was crucial in reaching MSM with education and focused prevention. To improve the effectiveness of the education, the contents and materials should be carefully reviewed and updated.

Limitations of this study should be noted. First, the impacts of the intervention programs could not be fully evaluated in the absence of baseline data, and comparisons of the outcome indicators were made using data from the midterm and endline surveys. In addition, the absence of data from a control group also made this impact evaluation difficult; it is possible that the reported effects of the interventions were due to external factors for which this study could not control. This is particularly relevant given the two-year gap between the midterm and endline surveys. Second, the findings might be limited by the unknown validity and reliability of the tools used in this study. To address this concern, we adapted items from previous studies in the same population to develop the questionnaire, and the questionnaire was carefully reviewed by experts in this area and pretested before the final version was constructed. Third, the use of self-reported measures may lead to desirability bias potential for both underreporting and over-reporting in the variables. It is likely that HIV risk behaviors and outcomes such as STI symptoms reported by the respondents in this study were under-estimated given the cultural norms governing sexual behaviors in Cambodia. The final limitation is the possibility of recall bias as the participants were asked to remember events that had taken place over the past several months.

## Conclusions

Findings of this evaluation suggest that participation in the SAHACOM project was associated with improvements in several sexual behaviors and health behavior outcomes such as reduction in the number of sexual partners, involvement in sexual intercourse with male commercial sex workers, and prevalence of STI symptoms and illicit drug use among MSM in this study. The results also suggest that the SAHACOM programs improved healthcare-seeking behaviors of those who experienced STI symptoms. However, the programs could not appreciably increase the rates of condom use in all types of relationships and HIV testing. Future studies should explore the specific barriers to condom use and HIV testing and intervention programs tailored to address these issues among MSM in Cambodia.

## Additional file

Additional file 1: Survey questionnaires. (DOCX 65 kb)

## Abbreviations

AIDS: Acquired immunodeficiency syndrome
ART: Antiretroviral therapy
C/PITC: Community/peer-initiated testing and counseling
CI: Confidence interval
HIV: Human immunodeficiency virus
NCHADS: National Center for HIV/AIDS, Dermatology, and STD
NGO: Non-governmental organization
OR: Odds ratio
SAHACOM: Sustainable Action against HIV and AIDS in Communities
SD: Standard deviation
STIs: Sexually transmitted infections
USAID: United States Agency for International Development
VCCT: Voluntary confidential counseling and testing
