# Supplementary material for: Improving prevention and care for HIV and sexually transmitted infections among men who have sex with men in Cambodia: the sustainable action against HIV and AIDS in communities (SAHACOM)
Source: BMC Health Serv Res. 2016 Oct 21;16:599. doi: 10.1186/s12913-016-1857-9 (PMC5073448; doi:10.1186/s12913-016-1857-9)
Supplement: Additional file 1: — Survey questionnaires. (DOCX 65 kb) [file 12913_2016_1857_MOESM1_ESM.docx]

**Appendix 4: Questionnaire for MSM/TG**

Questionnaire number

[Introduction: The following is to be read by the interviewer to the respondent]

My name is………………………………………..from KHANA. We are conducting a survey to evaluate the impacts of the SAHACOM Project. We would like to learn more about MSM/TG in regards to the results of HIV/AIDS program, their health risks, access to health care as well as their physical and mental health. The results from the survey will help us evaluate the effectiveness of what we have done in the past five years. We would like to request for your cooperation for about 30-45 minutes for an interview. Some of these questions are personal. However, you are free to refuse or discontinue the participation at any time without any consequences. All your answers will be absolutely confidential. I do not know your name, and there is no way that anyone can learn how you answered these questions. Please be truthful in your responses. Your participation is very important for the development of effective interventions and provision of supports for you and other people like yourself.

For more information or queries, please contact Dr. Yi Siyan, Research Director, KHANA:

Mobile: 012-417-170 | Landline: 023-211505, Ext.303| Fax: 023-214-049

Mailing: No. 33, Street 71, Phnom Penh, Cambodia, P.O Box. 2311 Phnom Penh 3

E-mail: [ysiyan@khana.org.kh](mailto:ysiyan@khana.org.kh)

Note: For interview of MSM/TG aged 15 and older

Do you agree to be interviewed? 0. No 1. Yes

Signature of the interviewer as a proof of receiving verbal consent from participant

…………………………………………………………………. Date……...……..……………………...

Did the interviewee abandon the interview? 0. No Yes (Specify question number………..)

Supervisor’s name……………………………………………… Date: …………………………………..

Data Entry Clerk 1: .…………………………………………… Date: …………………………………..

Data Entry Clerk 2: ……………………………………………. Date: …………………………………..

Provincial Code: 1. Battambang 2. Siem Reap

**Section 1: Socio demographic characteristics and general health**

SE1. How old are you? …………………Years

SE2. How do you regard yourself in terms of your own gender identity? 1. Male 2. Female 3. Both

SE3. What is your current relationship status? 1. Never married 2. Married and living together

3. Married but not currently living together 4. Not married but living with a partner

5. In a relationship but not living together 6. Divorced/separate/widow 7. Other (specify)

SE4. How many years of formal schooling have you completed? …………………Years

SE5. What is your main occupation?

1. Unemployment 2. Student 3. Motor/taxi diver

4. Farmer 5. Laborer 6. Self-employed business

7. Office worker (government, private company, NGO) 8.Uniformed officer (policeman, soldier)

9. Other (specify)……….

SE6. In the past 6 months, in average, how much money have you earned per month? ………………. Riel

SE7. With whom are you currently living? 1. Parents 2. Relative 3. Spouse 4. Friend 5. Sexual partner 6. Siblings 7. Alone 8. In an orphanage 9. Other (specify)

SE8. How long have you lived in this city? ……………. Months (1 if the answer is 1 month or less)

SE9. What type of accommodation are you currently living in? 1. Own home 2. Rented home

3. Someone’s home 4. Orphanage 5. No home 6. Other (specify)

SE10. In general, how would you rate your overall health?

1. Very good 2. Good 3. Neither good nor poor 4. Poor 5. Very poor

SE11. In general, how would you rate your overall quality of life?

1. Very good 2. Good 3. Neither good nor poor 4. Poor 5. Very poor

SE12. Have you ever thought about ending your life? 0. No 1. Yes

SE13. Have you ever attempted to end your life? 0. No 1. Yes

**Section 2. HIV/AIDS and STI**

HIV1. Have you ever been tested for HIV? 0. No (go to HIV7) 1. Yes

HIV2. During the past 6 months, have you been tested for HIV? 0. No 1. Yes

HIV3. Where did you get your most recent HIV test? 1. C/PITC 2. Finger prick 3. VCCT

4. Private hospital/clinic/laboratory 5. Public health center/hospital 6. Other (specify)

HIV4. Who advised you to get the most recent HIV test? 1. By myself 2. Boss/manager

3. Friends/colleagues 4. Family/relatives 5. Peer educator/NGO’s staff 6. Other (specify)

HIV5. Did you receive the result of your most recent HIV test? 0. No 1. Yes

HIV6. Did you receive HIV counseling when you received your most recent result? 0. No 1. Yes

HIV7. If you have not been tested, what was the main reason for not willing to get tested? 0. Been tested

1. No information about where to go 2. Didn’t think I am at risk for HIV 3. Feeling scared of the test

4. Feeling scared of positive result 5. Concerned about confidentiality 6. Concerned about stigma/discrimination 7. Other (specify)

HIV8. During the past 12 months, have you received any HIV education? 0. No (go to HIV10) 1. Yes

HIV9. During the past 12 months, what were the main sources of HIV education you have received? (Multiple answers allowed) 1. Media (TV/radio/newspaper) 2. Poster/billboard/booklet 3. Peer educator/outreach 4. Counseling at VCCT 5. Health staff at public facility 6. Other (specify)

HIV10. How do you regard yourself in terms of HIV infection risk compared to general people?

1. Much higher 2. Higher 3. Same 4. Lower 5. Much lower 6. Don’t know

HIV11. During the past 6 months, have you been diagnosed with an STI? 0. No (go to next section) 1. Yes

HIV12. Did you receive any treatment for your most recent STI? 0. No (go to next section) 1. Yes

HIV13. Where did you receive the treatment for your most recent STI?

1. Public HC/clinic/RH 2. Pharmacy 3. NGO clinic/hospital

4. Private clinic/hospital 5. Traditional healer 6. Other (specify)

**Section 3. Risky sexual behavior**

RSB1. Have you ever had sexual intercourse with a man or a woman? 0. No (go to RSB5) 1. Yes

RSB2. During the past 3 months, with how many partners have you had sexual intercourse? .......................

RSB3. During the past 3 months, how often have you used condom when you had sexual intercourse with men or women? 1. Always 2. Most of the time 3. Sometimes 4. Rarely 5. Never

RSB4. Did you use a condom in your most recent sexual intercourse? 0. No 1. Yes

RSB5. During the past 12 months, have you had a girlfriend? 0. No (go to RSB10) 1. Yes

RSB6. During the past 3 months, have you had sex with your girlfriend? 0. No (go to RSB 10) 2. Yes

RSB7. During the past 3 months, with how many girlfriends have you had sexual intercourse? ..................

RSB8. During the past 3 months, how often have you used condom when you had sex with your girlfriends?

1. Always 2. Most of the time 3. Sometimes 4. Rarely 5. Never

RSB9. The last time you had sex with your girlfriend, did you use condom? 0. No 3. Yes

RSB10. During the past 3 months, have you had a boyfriend? 0. No (go to RSB17) 1. Yes

RSB11. During the past 3 months, have you had sex with your boyfriend? 0. No (go to RSB 18) 1. Yes

RSB12. During the past 3 months, with how many boyfriends have you had sexual intercourse? ...................

RSB13. During the past 3 months, how often have you used condom with your boyfriend?

1. Always 2. Most of the time 3. Sometimes 4. Rarely 5. Never

RSB14. The last time you had sex with your boyfriend, did you use condom? 0. No 1. Yes

RSB15. During the past 3 months, have you had anal sex with your boyfriend?

0. No (go to RSB 17) 1. Yes

RSB16. During the past 3 months, how often have you used condom when you had anal sex with your boyfriend? 1. Always 2. Most of the time 3. Sometimes 4. Rarely 5. Never

RSB17. The last time you had anal sex with your boyfriend, did you use condom? 0. No 1. Yes

RSB18. During the past 3 months, have you had sex with a female commercial sex worker?

0. No (go to RSB 22) 1. Yes

RSB19. During the past 3 months, with how many female commercial sex workers have you had sex? ..........

RSB20. During the past 3 months, how often have you used condom when you had sex with female commercial sex workers? 1. Always 2. Most of the time 3. Sometimes 4. Rarely 5. Never

RSB21. The last time you had anal sex with a female commercial sex worker, did you use condom?

0. No 1. Yes

RSB22. During the past 3 months, ​have you had sex with a male commercial sex worker?

0. No (go to RSB 26) 1. Yes

RSB23. During the past 3 months, with how many male commercial sex workers have you had sex? .............

RSB24. During the past 3 months, how often have you used condom when you had sex with male commercial sex workers? 1. Always 2. Most of the time 3. Sometimes 4. Rarely 5. Never

RSB25. The last time you had sex with a male commercial sex worker, did you use condom? 0. No 2. Yes

RSB26. During the past 3 months, have you ever had sex with a woman in exchange for money or gifts?

1. No (go to RSB 30) 2. Yes

RSB27. During the past 3 months, with how many women have you had sex in exchange for money or gifts?

RSB28. During the past 3 months, how often have you used condom when you had sex with women in exchange for money or gifts? 1. Always 2. Most of the time 3. Sometimes 4. Rarely 5. Never

RSB29. The last time you had sex with a woman in exchange for money or gifts, did you use condom? 0. No 1. Yes

RSB30. During the past 3 months, have you had sex with a man in exchange for money or gifts?

0. No (go to the next section) 1. Yes

RSB31. During the past 3 months, with how many men have you had sex in exchange for money or gifts? ....

RSB32. During the past 3 months, how often have you used condom when you had sex with men in exchange for money or gifts? 1. Always 2. Most of the time 3. Sometimes 4. Rarely 5. Never

RSB33. The last time you had sex with a man in exchange for money or gifts, did you use condom?

0. No 1. Yes

RSB34. During the past 3 months, have you had anal sex with men in exchange for money or gifts?

0. No 1. Yes

RSB35. During the past 3 months, how often have you used condom when you had anal sex with men in exchange for money or gifts? 1. Always 2. Most of the time 3. Sometimes 4. Rarely 5. Never

RSB36. During the past 3 months, how often have you used lubricant when you had anal sex with men in exchange for money or gifts? 1. Always 2. Most of the time 3. Sometimes 4. Rarely 5. Never

**Section 4. HIV testing attitudes**

TA1. Getting tested for HIV helps people feel better. 1. Agree 2. Disagree

TA2. Getting tested for HIV helps people from getting HIV. 1. Agree 2. Disagree

TA3. People in my life would leave me if I had HIV. 1. Agree 2. Disagree

TA4. People who test HIV positive should hide it from others. 1. Agree 2. Disagree

TA5. I would rather not know if I have HIV. 1. Agree 2. Disagree

**Section 5. HIV/AIDS-related knowledge**

K1. Is AIDS spread by kissing? 1. No 2. Yes 3. DK

K2. Can a person get AIDS by sharing bathrooms with someone with HIV? 1. No 2. Yes 3. DK

K3. Can men give HIV to women? 1. No 2. Yes 3. DK

K4. Can women give HIV to men? 1. No 2. Yes 3. DK

K5. Must a person have many different partners to get HIV? 1. No 2. Yes 3. DK

K6. Can you get HIV by touching someone with HIV? 1. No 2. Yes 3. DK

K7. Does washing after sex help protect against HIV? 1. No 2. Yes 3. DK

K8. Is AIDS caused by spirits/supernatural forces? 1. No 2. Yes 3. DK

K9. Can a pregnant woman give AIDS to her baby? 1. No 2. Yes 3. DK

K10. Can a person get rid of AIDS by having sex with a virgin? 1. No 2. Yes 3. DK

K11. Is HIV the virus that causes AIDS? 1. No 2. Yes 3. DK

K12. Is there a cure for AIDS? 1. No 2. Yes 3. DK

**Section 6. Substance use**

SU1. During the past 3 months, have you drunk at least a full glass of any kinds of alcohol?

0. No (go to SU5) 1. Yes

SU2. How old were you when you drank at least a full glass of alcohol for the first time? ………….. Years

SU3. During the past month, on how many days have you been drunk? …………. Days

SU4. How do you regard yourself in terms of alcohol drinking?

0. Non-drinker 1. Social drinker 2. Heavy drinker

SU5. In your lifetime, have you smoked at least 100 cigarettes? 0. No (go to SU8) 1. Yes

SU6. In the past 3 months, on an average day, how many cigarettes have you smoked? ................. Cigarettes

SU7. How old were you when you smoked at least a whole cigarette for the first time? ………….Years

SU8. In the past 3 months, have you used any kind of illicit drugs (methamphetamine, heroin, ecstasy, inhalants, cocaine, or marijuana)? 0. No (go to next section) 1. Yes

SU9. During the past 3 months, what kind of illicit drugs have you used? 1. Methamphetamine (Yama, Yaba, Ice) 2. Injecting drugs (heroin) 3. Ecstasy 4. Sniffed glue 5. Marijuana 6. Other (Specify)

SU10. How old were you when you tried any kind of illicit drugs for the first time? …………. Years

SU11. In the past 3 months, how often have you used drugs?

1. A few times per month or less 2. A few times per week 3. Most of the day 4. Everyday

SU12. During the past 3 months, has anyone forced you to use illicit drugs? 1. No 2. Yes

SU13. What led you to try illicit drugs for the first time? 1. I tried it by myself 2. Someone gave it to me

3. Someone forced me to take it 4. I tried it with friends/colleagues 5. Other (specify)

SU14. During the past 3months, have you used injecting drugs? 0. No (go to section 5) 1. Yes

SU15. The last time you injected drugs, did you share needles or syringes with someone else?

0. No 1. Yes

**Section 7. General health questionnaire (GHQ-12)**

GHQ1. Have you recently been able to concentrate on what you are doing?

0. Better than usual 1. Same as usual 2. Less than usual 3. Much less than usual

GHQ2. Have you recently lost much sleep over worry?

0. Not at all 1. No more than usual 2. Rather more than usual 3. Much more than usual

GHQ3. Have you recently felt that you are playing a useful part in things?

0. More so than usual 1. Same as usual 2. Less so than usual 3. Much less than usual

GHQ4. Have you recently felt capable of making decisions about things?

0. More so than usual 1. Same as usual 2. Less than usual 3. Much less than usual

GHQ5. Have you recently felt constantly under strain?

0. Not at all 1. No more than usual 2. Rather more than usual 3. Much more than usual

GHQ6. Have you recently felt you couldn’t overcome your difficulties?

0. Not at all 1. No more than usual 2. Rather more than usual 3. Much more than usual

GHQ7. Have you recently been able to enjoy your normal day-to-day activities?

0. More so than usual 1. Same as usual 2. Less so than usual 3. Much less than usual

GHQ8. Have you recently been able to face up to your problems?

0. not at all 1. Not more than usual 2. Rather more than usual 3. Much more than usual

GHQ9. Have you recently been feeling unhappy or depressed?

0. Not at all 1. No more than usual 2. Rather more than usual 3. Much more than usual

GHQ10. Have you recently been losing confidence in yourself?

0. Not at all 1. No more than usual 2. Rather more than usual 3. Much more than usual

GHQ11. Have you recently been thinking of yourself as a worthless person?

0. Not at all 1. No more than usual 2. Rather more than usual 3. Much more than usual

GHQ12. Have you recently been feeling reasonably happy, all things considered?

0. not at all 1. Not more than usual 2. Rather more than usual 3. Much more than usual

**Section 8. Adverse childhood experiences**

ACE1. When I was growing up, I was hit or punished with a belt, a board, a cord, or other hard objects that hard enough to seek for medical care.

1. Never 2. Rarely 3. Sometimes 4. Often 5. Very often

ACE2. When I was growing up, people in my family said hurtful or insulting things to me.

1. Never 2. Rarely 3. Sometimes 4. Often 5. Very often

ACE3. When I was growing up, someone tried to touch me or make me touch them in a sexual way, make me do or watch sexual things, or did something sexual with me.

1. Never 2. Rarely 3. Sometimes 4. Often 5. Very often

ACE4. When I was growing up, I knew that there was someone to take care of me, protect me, and take me to medical care when got sick. 1. Never 2. Rarely 3. Sometimes 4. Often 5. Very often

ACE5. When I was growing up, there was someone in my family who helped me feel that I was loved and important. 1. Never 2. Rarely 3. Sometimes 4. Often 5. Very often

**Section 9. Family Dysfunction**

FD1. Has one of your parents or guardians been sometimes, often, or very often pushed, grabbed, slapped, had something thrown at her, kicked, bitten, or hit? 0. No 1. Yes

FD2. Has anyone in your family been a problem drinker or alcoholic or drug user? 0. No 1. Yes

FD3. Has anyone in your family been depressed or mentally ill or attempted suicide? 0. No 1. Yes

FD4. Have your parents ever been separated or divorced? 0. No 1. Yes

FD5. Has anyone in your family been to prison? 0. No 1. Yes

Thank You!
